# Concept, Design and Implementation of a Cardiovascular Gene-Centric 50 K SNP Array for Large-Scale Genomic Association Studies

**DOI:** 10.1371/journal.pone.0003583

**Published:** 2008-10-31

**Authors:** Brendan J. Keating, Sam Tischfield, Sarah S. Murray, Tushar Bhangale, Thomas S. Price, Joseph T. Glessner, Luana Galver, Jeffrey C. Barrett, Struan F. A. Grant, Deborah N. Farlow, Hareesh R. Chandrupatla, Mark Hansen, Saad Ajmal, George J. Papanicolaou, Yiran Guo, Mingyao Li, Stephanie DerOhannessian, Paul I. W. de Bakker, Swneke D. Bailey, Alexandre Montpetit, Andrew C. Edmondson, Kent Taylor, Xiaowu Gai, Susanna S. Wang, Myriam Fornage, Tamim Shaikh, Leif Groop, Michael Boehnke, Alistair S. Hall, Andrew T. Hattersley, Edward Frackelton, Nick Patterson, Charleston W. K. Chiang, Cecelia E. Kim, Richard R. Fabsitz, Willem Ouwehand, Alkes L. Price, Patricia Munroe, Mark Caulfield, Thomas Drake, Eric Boerwinkle, David Reich, A. Stephen Whitehead, Thomas P. Cappola, Nilesh J. Samani, A. Jake Lusis, Eric Schadt, James G. Wilson, Wolfgang Koenig, Mark I. McCarthy, Sekar Kathiresan, Stacey B. Gabriel, Hakon Hakonarson, Sonia S. Anand, Muredach Reilly, James C. Engert, Deborah A. Nickerson, Daniel J. Rader, Joel N. Hirschhorn, Garret A. FitzGerald

**Affiliations:** 1 The Institute for Translational Medicine and Therapeutics, School of Medicine, University of Pennsylvania, Philadelphia, Pennsylvannia, United States of America; 2 Broad Institute of Harvard and Massachusetts Institute of Technology, Cambridge, Massachusetts, United States of America; 3 Divisions of Genetics, Endocrinology & Program in Genomics, Children's Hospital, Boston, Massachusetts, United States of America; 4 Scripps Genomic Medicine, La Jolla, California, United States of America; 5 Department of Genome Sciences, University of Washington, Seattle, Washington, United States of America; 6 MRC SGDP Centre, Institute of Psychiatry, London, United Kingdom; 7 The Center for Applied Genomics, Division of Human Genetics, Department of Pediatrics, The Childrens' Hospital of Philadelphia, Philadelphia, Pennsylvania, United States of America; 8 The Wellcome Trust Centre for Human Genetics, University of Oxford, Oxford, United Kingdom; 9 The Cardiovascular Institute, University of Pennsylvania, Philadelphia, Pennsylvania, United States of America; 10 Illumina Incorporated, San Diego, California, United States of America; 11 Division of Prevention and Population Sciences, National Heart, Lung, and Blood Institute, Bethesda, Maryland, United States of America; 12 Beijing Genomics Institute at Shenzhen, Shenzhen, China; 13 Division of Genetics, Department of Medicine, Brigham and Women's Hospital, Harvard Medical School, Boston, Massachusetts, United States of America; 14 Medical Genetics Institute, Cedars-Sinai Medical Center, Los Angeles, California, United States of America; 15 Department of Medicine, University of California Los Angeles, Los Angeles, California, United States of America; 16 Institute of Molecular Medicine, University of Texas Health Science Center at Houston, Houston, Texas, United States of America; 17 Department of Clinical Science Malmoe, Diabetes and Endocrinology, Lund University, Sweden and Department of Medicine, Helsinki University Hospital, Helsinki, Finland; 18 Department of Biostatistics, University of Michigan, Ann Arbor, Michigan, United States of America; 19 Leeds Institute of Genetics, Health & Therapeutics, University of Leeds, Leeds, United Kingdom; 20 Diabetes Genetics Group, Institute of Biomedical and Clinical Science, Peninsula Medical School, Exeter, United Kingdom; 21 Department of Genetics, Harvard Medical School, Boston, Massachusetts, United States of America; 22 Department of Clinical Pharmacology, Barts and The London, Charterhouse Square, London, United Kingdom; 23 Department of Haematology and NHS Blood and Transplant, University of Cambridge, Cambridge, United Kingdom; 24 William Harvey Research Institute, Barts and The London, Charterhouse Square, London, United Kingdom; 25 Human Genetics Center, University of Texas Health Science Center, Houston, Texas, United States of America; 26 Department of Cardiovascular Sciences, University of Leicester, Leicester, United Kingdom; 27 Rosetta Inpharmatics, LLC, Merck & Co. Inc., Seattle, Washington, United States of America; 28 V.A. Medical Center and the University of Mississippi Medical Center, Jackson, Mississippi, United States of America; 29 Department of Internal Medicine II - Cardiology, University of Ulm Medical Center, Ulm, Germany; 30 Cardiology Division, Massachusetts General Hospital, Boston, Massachusetts, United States of America; 31 Department of Medicine and Clinical Epidemiology and Biostatistics, Population Genomics Program, McMaster University, Hamilton Health Sciences, Hamilton General Hospital, Hamilton, Ontario, Canada; 32 Departments of Medicine and Human Genetics, McGill University, Montréal, Québec, Canada; 33 Departments of Epidemiology and Biostatistics, Harvard School of Public Health, Boston, Massachusetts, United States of America; 34 Oxford Center for Diabetes, Endocrinology and Metabolism and Oxford NIHR Biomedical Research Center, Churchill Hospital, University of Oxford, Oxford, United Kingdom; 35 Department of Microbiology, Immunology and Molecular Genetics, University of California Los Angeles School of Medicine, Los Angeles, California, United States of America; 36 Genome Québec Innovation Centre, Montréal , Québec, Canada; Leiden University Medical Center, Netherlands

## Abstract

A wealth of genetic associations for cardiovascular and metabolic phenotypes in humans has been accumulating over the last decade, in particular a large number of loci derived from recent genome wide association studies (GWAS). True complex disease-associated loci often exert modest effects, so their delineation currently requires integration of diverse phenotypic data from large studies to ensure robust meta-analyses. We have designed a gene-centric 50 K single nucleotide polymorphism (SNP) array to assess potentially relevant loci across a range of cardiovascular, metabolic and inflammatory syndromes. The array utilizes a “cosmopolitan” tagging approach to capture the genetic diversity across ∼2,000 loci in populations represented in the HapMap and SeattleSNPs projects. The array content is informed by GWAS of vascular and inflammatory disease, expression quantitative trait loci implicated in atherosclerosis, pathway based approaches and comprehensive literature searching. The custom flexibility of the array platform facilitated interrogation of loci at differing stringencies, according to a gene prioritization strategy that allows saturation of high priority loci with a greater density of markers than the existing GWAS tools, particularly in African HapMap samples. We also demonstrate that the IBC array can be used to complement GWAS, increasing coverage in high priority CVD-related loci across all major HapMap populations. DNA from over 200,000 extensively phenotyped individuals will be genotyped with this array with a significant portion of the generated data being released into the academic domain facilitating *in silico* replication attempts, analyses of rare variants and cross-cohort meta-analyses in diverse populations. These datasets will also facilitate more robust secondary analyses, such as explorations with alternative genetic models, epistasis and gene-environment interactions.

## Introduction

**Figure 1:**
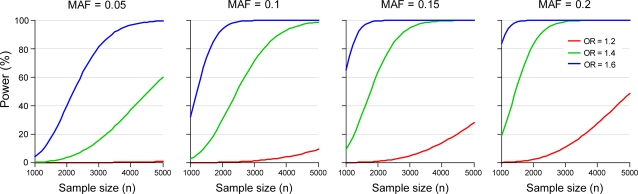
Power to detect main effect with 550 K SNPs using various case control sizes & MAFs. Genome wide association power calculated based on *n* unrelated cases and *n* unrelated controls. The disease model is assumed to be multiplicative with disease minor allele frequency (MAF) = 0.05, 0.1, and 0.2, and the odds ratio = 1.2, 1.4, and 1.6. Significance is assessed at the 5% level using Bonferroni correction assuming 550 K tests.

Cardiovascular disease (CVD), the leading cause of death in the developed world [1], has been shown to have significant heritability [2]–[6]. The pattern of CVD in developed countries has changed as the detection and management of risk factors such as hypertension, hypercholesterolemia and predisposition to thrombosis has coincided with a decline in the incidence of myocardial infarction (MI) and stroke [7]. Efforts to discover genetic determinants of complex disease have included analyses of genetic variation, using SNPs, between populations of individuals differing in incident or prevalent disease traits and/or clinical events. However, many apparent associations have not replicated for reasons including inadequate sample size, imprecise or inaccurate phenotyping, insufficiently stringent statistical thresholds, genuine heterogeneity of causality and population stratification [8], [9]. The International HapMap Project [10], combined with advances in genotyping technologies, has led to the generation of multiple array-based SNP genotyping products for GWAS. These developments enable reasonably dense and unbiased global scans of the human genome which have already identified novel loci associated with CVD [11]–[14]. Despite the value of the GWAS approach, a number of limitations exist, including cost and incomplete coverage in the HapMap samples. GWAS also have relatively low power to detect subtle, but potentially important effects, in studies of “typical” sample sizes. For example, calculations of the general power to detect a primary effect using an array with >500 K SNPs are depicted in Figure 1.

Array-based genotyping technologies that have enabled GWAS also permit flexibility in choosing the scope and density of SNPs for candidate gene studies. For example, they allow improved exploitation of recent deep resequencing data, enabling more accurate capture of genetic diversity across populations [15]. Such custom platforms, at scale, allow inclusion of large numbers of plausible candidate loci with a marginal increase in cost.

We describe here the design and implementation of a custom 50 K SNP genotyping array, primarily aimed at assaying SNPs in candidate genes and pathways for cardiovascular, inflammatory and metabolic phenotypes. Design of this genotyping array was led by investigators from the Institute of Translational Medicine and Therapeutics (ITMAT), the Broad Institute and by the National Heart Lung and Blood Institute (NHLBI) supported Candidate-gene Association Resource (CARe) Consortium. The custom SNP array is hereafter referred to as the “IBC array” (ITMAT-Broad-CARe array). A consortium of international academic and industrial partners have committed to using the IBC array to genotype DNA from more than 200,000 individuals who have been extensively phenotyped for risk factors and clinical evidence of vascular disease. The objectives of forming this consortium were (i) to pool expertise for selection of both loci and SNPs; (ii) to reduce costs by producing a standardized genotyping platform; and (iii) to facilitate cross cohort meta-analyses for a large set of SNPs in high priority candidate loci. Here we formally describe the resource and assess coverage of the genetic variants from prioritized loci generated on the SNP panel with the HapMap populations. We also evaluate the coverage of the array with the major GWAS products.

## Methods

### Processes for Loci selection

We used the following search string in PubMed ‘(genotype OR snp OR allele* OR polymorphism OR variant) AND (coronary OR heart OR myocardial OR cardiac OR ischemic OR hypertension OR thrombosis) AND (linkage OR association OR control OR randomized OR trial)’ covering publications from 1978 to May 2007, for version1 of the IBC array (IBCv1), and to October 1^st^ 2007 for the 2^nd^ version of the IBC array (IBCv2). Key information was collated including: PMID number, publishing journal, size and population examined, loci and SNPs studied (including the respective rs numbers, where retrievable) and functional evidence. Over 2,400 published studies were systematically analyzed. Emphasis was placed on sample size, data quality and strength of the described associations. Genes with known of putative association with phenotypes for sleep, lung, and blood diseases were also nominated. Input was also solicited directly from investigators within and outside the consortium.

Several pathway-based tools were used to identify additional biologically plausible candidate genes: Kyoto Encyclopedia of Genes and Genomes (KEGG), [16]; Protein ANalysis THrough Evolutionary Relationships (PANTHER), (www.pantherdb.org) and BioCarta (www.biocarta.com). These tools were employed to collate additional genes from key pathways including lipid metabolism, thrombogenesis, circulation and gas exchange, insulin resistance, metabolism, and inflammation, oxidative stress and apoptosis.

Early access was provided to a number of unpublished mouse atherosclerosis expression quantitative trait loci (eQTL) datasets. Genes predicted to be causal for atherosclerotic lesion size in genetic crosses of mice with differing susceptibility to atherosclerosis were identified [17] based on (i) the correlation between transcript levels and lesion size, (ii) the overlap of expression and atherosclerosis QTLs and (iii) the likelihood of a causal rather than a dependent or reactive relationship based on Bayesian modeling.

Early access was provided to a number of key findings emerging from five GWAS:

- The Wellcome Trust Case Control Consortium (WTCCC) Hypertension study [18];
- The WTCCC type 2 diabetes (T2D) study [12], [18];
- The WTCCC coronary artery disease study, [18];
- Finland-United States Investigation of NIDDM Genetics (FUSION) study [19]; and
- The Broad-Novartis-Lund Diabetes Genetics Initiative (DGI), [11].

A three-way meta-analysis of the WTCCC, Broad-Novartis-Lund and FUSION studies led to the generation of stronger T2D candidate loci for inclusion on the custom array. We also included SNPs reaching genome-wide significance from the WTCCC Rheumatoid Arthritis, Crohn's Disease and Type-1 Diabetes studies [18].

Over 2,400 of the collated loci were placed on a database (http://bmic.upenn.edu/cvdsnp) along with key information displayed for each respective gene: the number of SNPs required to tag the four HapMap representative populations at various minor allele frequencies (MAFs) and r^2^ thresholds; SymAtlas® expression profiling for over 70 specific human tissues and cell-types [20]; links to National Center for Biotechnology Information (NCBI), Online Mendelian Inheritance in Man (OMIM) and other reference databases; public resequencing information; Jackson Lab Mouse (http://jaxmice.jax.org) and other phenotypic data. A voting system built into this database facilitated consensus amongst the consortium investigators for ranking genes proposed for inclusion on the IBC array. Over 2,000 of these loci were prioritized into three density criteria for tagging, as described below, based on voting by the participating investigators.

### Processes for SNP selection

**Group 1** (n = 435 loci); genes and regions with a high likelihood of functional significance, including established mediators of vascular disease, loci derived from GWAS and those shown to be associated with phenotypes of interest. Tag SNPs for these loci were selected to capture known variation with MAF>0.02 and an r^2^ of at least 0.8 in HapMap populations and SeattleSNPs where available (for formal description, see Calculation of Coverage section below).

**Group 2** (n = 1,349 loci); candidate loci that are potentially involved in phenotypes of interest or established loci that required very large numbers of tagging SNPs. SNPs for these loci were selected for MAFs>0.05 with an r^2^ of at least 0.5 in HapMap populations and SeattleSNPs where available.

**Group 3** (n = 232 loci); comprised mainly of the larger genes (>100 kb) which were of lower interest *a priori* to the consortium investigators. Only non-synonymous SNPs (nsSNPs) and known functional variants of MAF>0.01 were captured for these loci.

Assays for specific SNPs of known or putative functionality and those shown to be highly associated with vascular disease from literature searching were directly ‘forced’ into the array content, with the aim of facilitating more powerful downstream meta-analyses with previously published data. nsSNPs and known functional variants of MAF>0.01 were selected where possible for all genes of interest.

SNPs from Group 1 and 2 loci were first chosen using the TAGGER software [21]. Assays for SNPs in Group 1 loci were designed to be inclusive of the intronic, exonic, untranslated regions (UTRs) and 5 kb of the proximal promoter regions derived from NCBI build 35 with intronic, exonic and flanking UTRs covered for the ‘Group 2’ loci. This approach generated a set of tag SNPs and multimarker predictors that capture variation in the four HapMap populations (CEU, Centre d'Etude du Polymorphisme Humain collection; CHB, Han Chinese in Beijing, China; JPT, Japanese individuals from Tokyo, YRI, Yoruba from Ibadan, Nigeria; HapMap Data release 21/phase II July 2006 on NCBI build 35, dbSNP build 125). Where available, we also employed SeattleSNPs (http://pga.gs.washington.edu) and Environmental Genome Project (EGP), (http://egp.gs.washington.edu) resequencing data to identify additional tags, not represented in the HapMap populations, using ldSelect [22]. We choose SNPs that were observed at least twice in unrelated individuals.

SNPs were categorized by their assay design scores for the Infinium genomic platform technology (Illumina, CA), based on a theoretical algorithm and all previously attempted wet-lab Infinium assays. In an attempt to reduce the proportion of failed assays on the final product, we pre-filtered most SNPs for Infinium design scores > = 0.6, finding appropriate proxies where possible.

### Additional Probes

Two panels of ∼1,500 and ∼400 admixture and Ancestry Informative SNP markers (AIMs) were included for African versus European ancestry, and regional European (e.g. Northern versus Southern) ancestral populations respectively to enable admixture mapping and adjustment for population stratification in studies comprised of individuals from these ancestries. These SNPs were based on panels generated previously [23], [24], excluding SNPs failing Hardy-Weinberg equilibrium (*P*>0.01). The AIMs panels are listed within the IBC resource site (http://bmic.upenn.edu/cvdsnp/updates/ancestry_informative_markers-ibc-v1.xls). The incorporation of admixture and AIM panels enables admixture mapping in African Americans and adjustment for population stratification in African Americans and European Americans.

Genomic regions demonstrating ultra-high conservation across species were identified as previously described [25]. Briefly, regions were identified with at least 98% sequence similarity, with a minimum length of 200 nucleotides within human-mouse-dog, human-mouse-rat or human-chicken alignments. In addition, conserved regions with sequence identity of at least 95% near the Group 1, 2 and 3 loci were selected. All variants within these regions (n = 1023 SNPs), as evident in at least one HapMap population, were included on the IBC array.

### Genotyping Platform Final Probe Selection

Assays for 49,234 SNPs were attempted using the Infinium technology for IBCv1 [26], [27] which became available to consortium members in October 2007. Assays for an additional 4,050 SNPs were added to the initial content to comprise the IBCv2 array to be released in the Summer of 2008. The additional IBCv2 SNP content was mainly derived from the following:

- Proxy assays were attempted for a total of 596 SNPs that failed during the manufacture of IBCv1. Some 1,143 of the 3,769 SNPs that failed assay conversion in manufacturing were tagged by at least one passing SNP already on the array (CEU r^2^> = 0.8). Suitable proxy assays for the remaining 2,626 SNPs were prioritized for supplementation based on MAFs in the main HapMap populations and their respective r^2^ value and Infinium design scores;
- Additional literature scans for CVD genetic findings since completion of IBCv1 using the same Pubmed search criteria described earlier;
- Combined GWAS analyses of the Diabetes Genetics Initiative [11], the FUSION study [19] and the SardiNIA study [28] of low-density lipoprotein (LDL), high-density lipoprotein (HDL) and triglycerides. Analyses of these datasets were followed by targeted replication analyses in two independent efforts [29], [30]. Common SNPs at 19 loci, 12 of which showed previous association (and are represented on IBCv1), were shown to be reproducibly associated with levels of LDL, HDL and/or triglycerides. Two of the seven novel loci described (P<5×10^−8^ for each new locus) are associated with LDL, two with HDL and five with triglycerides;
- Schadt and colleagues performed global expression profiling on liver tissue and a GWAS on 427 Caucasian subjects. Genotyping of >750,000 SNPs was performed and SNPs were tested for association to each of the gene expression traits. We included 130 SNPs with MAF>0.04 from this study on IBCv2 that were identified as significantly associated with at least one gene expression trait (p-value for association <10^−9^; FDR∼1%), [31];
- Early access to ten novel loci relating to intermediate and continuous CVD-related traits identified from an additional meta-analyses of the FUSION [19], DGI [11] and WTCCC-T2D [12] studies and were replicated in independent samples with an effective sample size of up to 53,975 individuals [32].

### Minor Allele Frequency Estimates

MAFs were assessed across the IBCv1 arrays in 6067 DNA samples collated from three studies with five populations of self described ethnicity, screened for cardiovascular traits; Caucasians (n = 4244 European and n = 1054 US Caucasians); African Americans (n = 384) and South Asians (n = 385). All samples described were genotyped following approval by the relevant institutional review boards. In each respective population, the minor allele frequency for each SNP on the IBCv1 array was determined. Histograms were generated with various allele frequency bins to determine the distribution of allele frequencies in each population.

### Calculation of Coverage

We used previously described methods [33] to calculate coverage of HapMap SNPs. Briefly, pairwise r^2^ values were calculated using the expectation algorithm [34] based on the genotypes from HapMap release 22. Maximum r^2^ values were calculated for each SNP list (HapMap release 22) with each SNP on the array being tested. All pair-wise combinations were considered within 200 kb. For chromosome X, only female individuals were used; otherwise, all unrelated individuals were used.

## Results

### Formal analysis of assay conversions

Assays for 49,234 SNPs were attempted during manufacturing with 45,237 SNPs successfully passing the manufacturer's criteria. Reasons for failures included sub-optimal probe synthesis and insufficient resolving of assay traces, potentially due to nearby hidden SNPs or copy number variants (CNVs). Table 1 outlines the type of genetic variants contained on IBCv1. Table S1 shows the expected and observed conversion rates across the passing SNPs. Over 1,300 more SNPs failed than had been predicted by the theoretical conversion scores.

**Table 1:** Genetic variant types assayed on IBCv1.

Variant type	Number of SNPs (% of total)	Variant Subtype (number of SNPs)
Exonic	4476 (9.9)	
		Synonymous (825)
		Non-synonymous (3280)
		Other coding (371)
Intronic	29367 (64.9)	
Untranslated Regions (UTRs)	1602 (3.5)	
		5′ UTR (162)
		UTR (80)
		3′ UTR (1360)
Flanking UTRs	9792 (21.6)	
		Flanking 5′ UTR (5966)
		Flanking 3′ UTR (3826)
**Total**	**45237**	

Genetic variants covered on the IBC version 1 array categorized into exonix, intronic, untranslated regions (UTRs) or flanking UTRs.

### Quality control

DNA samples from 117 HapMap individuals were genotyped on the IBCv1. We have made these genotype files available for the community (http://bmic.upenn.edu/cvdsnp/updates/hapmap_qc_samples-illumina.xls). 37,431 (82.7%) SNPs are evident in HapMap. Approximately 99.5% concordance was observed against respective HapMap data across the 117 samples and 52 Mendelian inconsistencies were observed in 25 HapMap trios (Table S2). All inconsistencies were attributable to a deleted region from chromosome 1 in a proband which may be caused by a *bona fide de novo* micro-deletion event or an artifact of the DNA derived from the EBV-immortalised cell-lines. Complete reproducibility was observed across six replicate samples (Table S3).

### Comparisons of MAFs of IBCv1 across DNA samples from different populations

**Figure 2:**
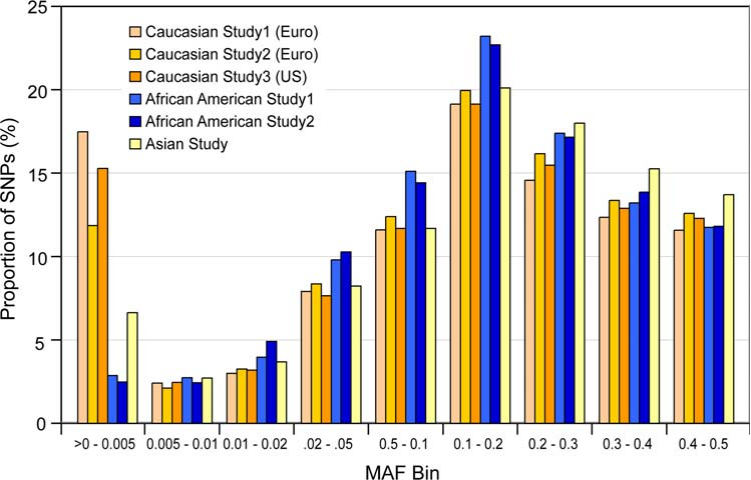
MAF distribution for IBCv1 assays across studies of Caucasians, African Americans and South Asians. Proportion of polymorphic variants (as a % of total) are described on the y-axis with the x-axis illustrating bins of the minor allele frequencies (MAF) for six different studies; Caucasian Study 1 (n = 2094 European); Caucasian Study 2 (n = 2150 European); Caucasian Study 3 (n = 1054 European American); African American Study 1 (n = 254); African American Study 2 (n = 130) and South Asians (n = 385).

MAFs were assessed across the IBCv1 arrays in 6067 DNA samples collated from three studies with five populations of self described ethnicity, screened for cardiovascular traits; Caucasians (n = 4244 European and n = 1054 US Caucasians); African Americans (n = 384); and South Asians (n = 385). Some 1415 assays across the complete dataset were monomorphic. 2705 and 2566 assays were, respectively, monomorphic across self-described Caucasians and African Americans. Figure 2 illustrates the distribution of MAFs in the Caucasian, African American and South Asian populations, respectively. The various bins for MAFs>0.01 were comparable across all populations examined. Significant variability was evident for variants with MAFs<0.005 which is expected, given the frequency of observations, the varying number of individuals in each ethnic group studied and the natural allele frequency differences of such variants across populations.

### Comparison of IBCv1 with the current GWAS products

**Figure 3:**
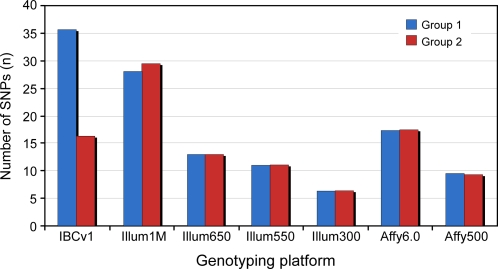
Average number of SNPs per Group 1 and 2 loci, respectively, on IBCv1 compared with the major GWAS products. IBCv1 Group 1 and Group 2 loci (n = 435 and 1,349) are captured at MAF>0.02, r^2^ 0.8 and MAF>0.05, r^2^ 0.5 in HapMap populations and SeattleSNPs, and illustrated in blue and red respectively. IBCv1 refers to the ITMAT-Broad- CARE version1 array, Illum650, Illum550 & Illum300 refer to Illumina HumanHap650Y, HumanHap550 and HumanHap300 Infinium products containing ∼660 K, ∼550 K & ∼317 K SNPs respectively and Affy 6.0 and Affy 5.0 refers to Affymetrix SNP array products containing ∼907 K & 500 K respectively.

The average number of SNPs across the Group 1 and Group 2 loci of IBCv1 were compared with several GWAS products (Figure 3). The average coverage for Group 1 loci is ∼36.5 SNPs per locus in IBCv1. The Illumina Human1M and Affymetrix 6.0 platform, for comparison, have an average of ∼28.0 and ∼17.4 SNPs respectively across the equivalent IBC loci. The average number of SNPs observed for the Group 2 loci is ∼16.3 SNPs which is comparable with the current GWAS products.

**Figure 4:**
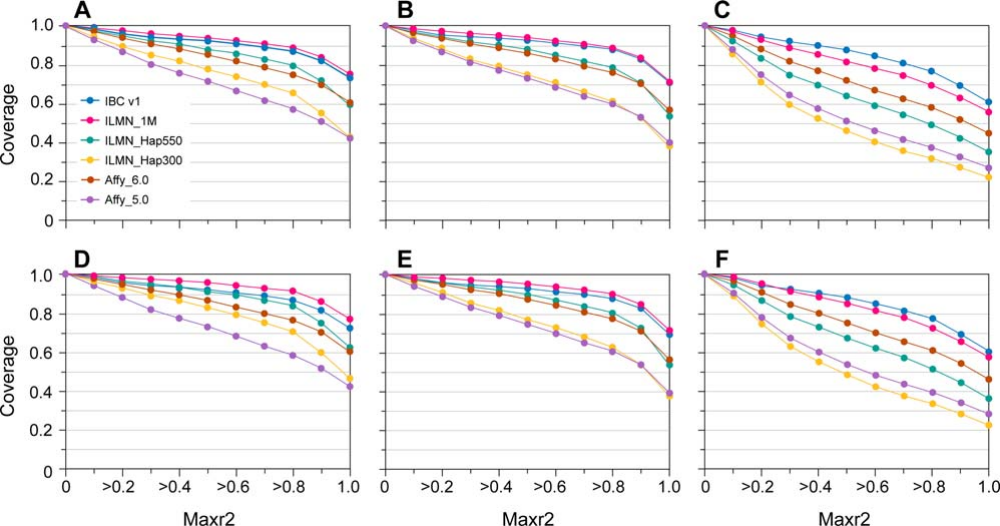
Coverage of IBCv1 versus GWAS products for Group1 loci in HapMap populations. Cumulative coverage (y axis) of the HapMap 22 release was assessed using Max r^2^ (× axis), at an MAF cutoff of 0.02, in CEPH (CEU) (A), Chinese (CHB) plus Japanese (JPT), (B) and Yoruba (YRI) HapMap individuals (C). Coverage was also assessed at an MAF cutoff of 0.05 in CEU (D), CHB+JPT (E), and YRI individuals (F). IBCv1 refers here to version1 ITMAT-Broad-CARe array using 45,237 SNPs passed by the manufacture. ILMN_1M, ILMNHap550 and ILMNHap300 refer to Illumina's Human1M, HumanHap550 (555,352 SNPs) and HumanHap300 (317,503 SNPs) products, respectively. Affy_6.0 and Affy_5.0 refers to Affymetrix 6.0 & Affymetrix 5.0 array products containing ∼906,600 SNPs & 500,568 SNPs respectively.

The coverage of HapMap SNPs was evaluated for all Group 1 loci against the various GWAS products in the HapMap individuals. The maximum r^2^ value was calculated between each HapMap SNP in the region to a SNP in each respective product. Figures 4 (a) through (f) shows the composite coverage of Group 1 loci from IBCv1 versus several GWAS products for CEU, YRI and CHB+JPT HapMap individuals using MAF cutoffs of >0.02 and >0.05 across the spectrum of r^2^ thresholds. The coverage using CEUs and CHB+JPT is comparable across all products, although the IBCv1 coverage for YRI is greater. A number of the GWAS products and the IBC array are strongly biased for composition of HapMap SNPs and will obviously have skewed coverage when directly compared. Over 20% of IBCv1 Group1 loci SNPs have not been assayed directly in HapMap with the majority of these additional SNPs derived from SeattleSNPs and the literature. Thus, the IBC array is likely to be more representative of broader population allelic architecture.

**Figure 5:**
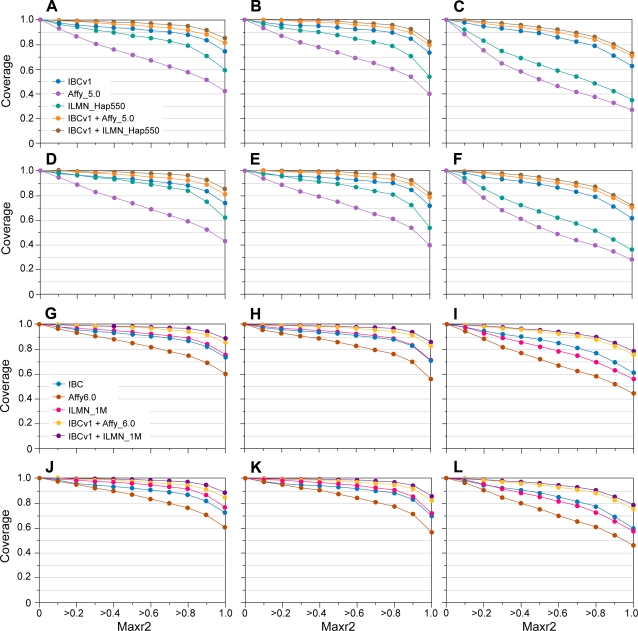
Combined coverage of IBCv1 with the 500 K and one million SNP products for Group1 loci in HapMap populations. For the combined coverage of IBCv1 with the 500 K SNP products (A–F): coverage (y axis) was assessed using Max r^2^ (× axis), at an MAF cutoff of 0.02, for the HapMap 22 release in CEPH (CEU) (A), Chinese (CHB) plus Japanese (JPT) (B), and Yoruba (YRI) HapMap individuals (C). Coverage was also assessed at an MAF cutoff of 0.05 in CEU (D), CHB+JPT (E), and YRI individuals (F). For combined coverage with the one million SNP products (G–L): Coverage (y axis) was also assessed using Max r^2^ (× axis), at an MAF cutoff of 0.02, for the HapMap 22 release in CEPH (CEU) (G), Chinese (CHB) plus Japanese (JPT) (H), and Yoruba (YRI) HapMap individuals (I) and coverage with an MAF cutoff of 0.05 in CEU (J), CHB+JPT (K), and YRI individuals (L). IBCv1 refers here to the version1 ITMAT-Broad-CARe array using 45,237 SNPs that passed manufacturers criteria. Affy_5.0 refers to the Affymetrix 5.0 array and ILMN_HapMap550 refers to the Infinium 550 K HumanHap array which contains 500,568 and 555,352 SNPs respectively. Affy_6.0 refers to the Affymetrix 6.0 array and ILMN_1M refers to the Illumina Infinium one million SNP array containing ∼906,600 and ∼1,050,000 SNPs respectively.

The combined coverage of IBCv1 with a number of the GWAS products was assessed for Group 1 loci. The coverage of the IBCv1 alone, with both of the 500 K SNP GWAS and with the one million SNP array products across the Group 1 loci is illustrated in Figure 5 under varying MAF thresholds across the HapMap populations. The combined coverage using IBCv1 with both 1 M SNP products is similar for Caucasians and Asian HapMap samples. The increase in coverage is more pronounced in African HapMap samples, reflecting the dense marker tagging for YRI in the IBC array.

## Discussion

We have produced a custom SNP array designed to capture genetic variation in prioritized loci known or postulated to increase risk of cardiovascular, metabolic and inflammatory diseases. Custom SNP selection allowed us to: (a) ensure selective and consistent coverage for a range of prioritized loci across multiple ancestries, (b) provide additional representative coverage to HapMap in loci of major interest, using SNP content from various sources including recent resequencing efforts; and (c) assay directly specific SNPs of interest such as those derived from previously published studies and known non-synonymous SNPs with MAF>0.01. The IBC array reveals greater depth of coverage than GWAS products with respect to information content and haplotype diversity in the high priority regions. This is particularly true of coverage for African HapMap representative samples. A modest fraction of tagging SNPs from the Group 1 loci on the IBC array are derived from SeattleSNPs analyses and were not assayed directly in HapMap, thus it is likely that the cumulative coverage of variation in these regions is actually underestimated in the current results. It is worth noting that as HapMap was predominantly used for the design of the IBC array (as well as many of the commercial products), then additional densely genotyped or sequenced populations, outside those covered in the original HapMap, would be required for a completely unbiased assessment of coverage.

Despite the recent reductions in price of whole genome SNP arrays, GWAS still remain expensive endeavors and power to cost issues are important factors in study design [35], [36]. When a two-stage GWAS design is employed, the need for custom genotyping in the second stage can increase costs per individual to a substantial fraction of the cost of the initial stage. GWAS are limited because the cost prohibits acquisition of the sample size needed to overcome the multiple testing problems inherent in gene-gene analyses. Generating a consistent set of genotypes in candidate genes within a large sample may in the short term provide a better balance between sample size and number of testable hypotheses than can be provided by the more expensive and extensive GWAS, and will likely permit a better-powered assessment of the contribution of epistasis to complex traits. Furthermore the rational selection and greater density of coverage in these prioritized loci in the IBC array biases towards detection of disease causing loci, that complements the discovery nature of an unbiased GWAS strategy. As the IBC array is available as a standard tool to the community, the cost is greatly reduced with respect to custom genotyping. The IBC array can clearly be used in conjunction with GWAS products to increase coverage in the high priority regions, permitting greater exploration of gene-interactions and other secondary analyses for the collated high priority loci.

The HapMap project had a bias towards discovery and genotyping of variants with MAFs>0.05, but over 40% of SNPs were observed to have MAFs<0.05 [10] and the ENCyclopedia Of DNA Elements (ENCODE) project indicates ∼60% of SNPs have MAFs<0.05 [37]. Many case-control association studies of complex diseases have tended to use MAFs>0.05 due to the power constraints of typical samples sizes. Gorlov and colleagues recently postulated that SNPs that are potentially deleterious are subjected to weak purifying selection and may represent significant contributors to genetic components of common disease [38]. Indeed potentially damaging nsSNPs are skewed toward rarer distribution in the HapMap project, ENCODE and SeattleSNPs. In a recent study comparing the Illumina 14.5 K nsSNP array with GWAS tools (Affy 5.0, Illumina HumanHap300 and HumanHap550), Evans and colleagues found that the GWAS products failed to capture most of the rare variants present on the nsSNP platform [39]. The major nsSNP studies attempted thus far have had modest sample sizes of ∼1500 cases and controls [40]. All nsSNPs>MAF 0.01 have been targeted in the design of the IBC array using information from both HapMap and SeattleSNPs and have tagged to MAFs>0.02 for a large number of key loci related to vascular diseases. Analyses of such lower frequency variants will be facilitated by the formation of an international consortium of investigators committed to using this platform. This will permit collaborative meta-analyses across a broad range of phenotypes. The CARe Consortium, for example, will make their IBCv2 genotype data (n∼50,000 samples) and most related phenotypic data available to the academic community.

The IBC array is one of the first disease-specific custom arrays with highly focused content to be used on a large scale. We anticipate further generations of the IBC array and that future aggregation of large cohorts and studies with similar disease traits will become commonplace, affording significant cost reductions and increased power to detect effects of modest size.

## Supporting Information

Table S1 Bins of SNPs with observed and expected Infinium conversion scores The distribution of SNPs binned according to Infinium score from 0.1 to 1 where a score of 0.8 indicates an 80% likelihood for conversion to a successful assay, 1.0 indicates an assay has ∼100% theoretical score etc. A value of 1.1 indicates that an Infinium assay for SNPs has previously been successful in manufacture and analyses. Percentages are indicated in brackets.
(0.05 MB DOC)

Table S2 Observed IBCv1 Mendelian consistency across 25 HapMap trios. Observed Parent-Parent-Child (PPC) heritability errors across the IBC version1 array using 25 HapMap individuals, where NA number denotes the official HapMap identifier.
(0.08 MB DOC)

Table S3 Observed replicate consistency using six HapMap individuals. Observed IBC version1 array genotyping errors for six replicate HapMap samples, where NA number denotes the official HapMap identifier.
(0.05 MB DOC)
