# Supplementary material for: Concept, Design and Implementation of a Cardiovascular Gene-Centric 50 K SNP Array for Large-Scale Genomic Association Studies
Source: PLoS One. 2008 Oct 31;3(10):e3583. doi: 10.1371/journal.pone.0003583 (PMC2571995; doi:10.1371/journal.pone.0003583)
Supplement: Table S1 — Bins of SNPs with observed and expected Infinium conversion scores The distribution of SNPs binned according to Infinium score from 0.1 to 1 where a score of 0.8 indicates an 80% likelihood for conversion to a successful assay, 1.0 indicates an assay has ∼100% theoretical score etc. A value of 1.1 indicates that an Infinium assay for SNPs has previously been successful in manufacture and analyses. Percentages are indicated in brackets. (0.05 MB DOC) [file pone.0003583.s001.doc]

SUPPORTING INFORMATION

| **Distribution bins of Infinium scored SNPs** | **>0.1 <0.5** | **>0.5 <0.6** | **>0.6 <0.7** | **>0.7 <0.8** | **>0.8 <0.9** | **>0.9 <0.99** | **1** | **1.1** | **Total** |
| --- | --- | --- | --- | --- | --- | --- | --- | --- | --- |
| SNP number in each bin | 213 | 219 | 1053 | 2042 | 6496 | 14714 | 11199 | 13298 | 49234 |
| Predicted failures per bin (%) | 143 (67.1) | 99 (45.2) | 367 (34.9) | 510 (25.0) | 831 (12.8) | 736 (5.0) | 0 (0) | 0 (0) | 2686 (5.5) |
| Actual failures per bin | 56 (26.3) | 37 (16.9) | 138 (13.1) | 220 (10.7) | 587 (9.0) | 1224 (8.3) | 870 (7.8) | 864 (6.5) | 3996 (8.1) |

**Table S1: Bins of SNPs with observed and expected Infinium conversion scores**

The distribution of SNPs binned according to Infinium score from 0.1 to 1 where a score of 0.8 indicates an 80% likelihood for conversion to a successful assay, 1.0 indicates an assay has ~100% theoretical score etc. A value of 1.1 indicates that an Infinium assay for SNPs has previously been successful in manufacture and analyses. Percentages are indicated in brackets.
