# Supplementary material for: Concept, Design and Implementation of a Cardiovascular Gene-Centric 50 K SNP Array for Large-Scale Genomic Association Studies
Source: PLoS One. 2008 Oct 31;3(10):e3583. doi: 10.1371/journal.pone.0003583 (PMC2571995; doi:10.1371/journal.pone.0003583)
Supplement: Table S2 — Observed IBCv1 Mendelian consistency across 25 HapMap trios. Observed Parent-Parent-Child (PPC) heritability errors across the IBC version1 array using 25 HapMap individuals, where NA number denotes the official HapMap identifier. (0.08 MB DOC) [file pone.0003583.s002.doc]

| **HapMap Parent1** | **HapMap Parent2** | **Offspring** | **Total no. of observed SNPs** | **SNP errors observed** | **P-P-C Heritability Frequency** |
| --- | --- | --- | --- | --- | --- |
| NA06993 | NA06985 | NA06991 | 45081 | 0 | 1 |
| NA06994 | NA07000 | NA07029 | 45100 | 0 | 1 |
| NA07357 | NA07345 | NA07348 | 45110 | 0 | 1 |
| NA11881 | NA11882 | NA10859 | 45127 | 0 | 1 |
| NA11992 | NA11993 | NA10860 | 45034 | 0 | 1 |
| NA12043 | NA12044 | NA10857 | 45097 | 0 | 1 |
| NA12146 | NA12239 | NA10847 | 45050 | 0 | 1 |
| NA12154 | NA12236 | NA10830 | 44986 | 0 | 1 |
| NA12264 | NA12234 | NA10863 | 45054 | 0 | 1 |
| NA12750 | NA12751 | NA12740 | 45096 | 0 | 1 |
| NA12812 | NA12813 | NA12801 | 45098 | 0 | 1 |
| NA12874 | NA12875 | NA12865 | 45055 | 52 | 0.9988 |
| NA12891 | NA12892 | NA12878 | 45062 | 0 | 1 |
| NA18516 | NA18517 | NA18515 | 45070 | 0 | 1 |
| NA18853 | NA18852 | NA18854 | 45069 | 0 | 1 |
| NA18856 | NA18855 | NA18857 | 45078 | 0 | 1 |
| NA19092 | NA19093 | NA19094 | 45093 | 0 | 1 |
| NA19119 | NA19116 | NA19120 | 45066 | 0 | 1 |
| NA19138 | NA19137 | NA19139 | 45097 | 0 | 1 |
| NA19141 | NA19140 | NA19142 | 45078 | 0 | 1 |
| NA19144 | NA19143 | NA19145 | 45090 | 0 | 1 |
| NA19160 | NA19159 | NA19161 | 45100 | 0 | 1 |
| NA19171 | NA19172 | NA19173 | 45083 | 0 | 1 |
| NA19192 | NA19193 | NA19194 | 45015 | 0 | 1 |
| NA19239 | NA19238 | NA19240 | 45012 | 0 | 1 |

**Table S2: Observed IBCv1 Mendelian consistency across 25 HapMap trios.**

Observed Parent-Parent-Child (PPC) heritability errors across the IBC version1 array using 25 HapMap individuals, where NA number denotes the official HapMap identifier.
