# Supplementary material for: Concept, Design and Implementation of a Cardiovascular Gene-Centric 50 K SNP Array for Large-Scale Genomic Association Studies
Source: PLoS One. 2008 Oct 31;3(10):e3583. doi: 10.1371/journal.pone.0003583 (PMC2571995; doi:10.1371/journal.pone.0003583)
Supplement: Table S3 — Observed replicate consistency using six HapMap individuals. Observed IBC version1 array genotyping errors for six replicate HapMap samples, where NA number denotes the official HapMap identifier. (0.05 MB DOC) [file pone.0003583.s003.doc]

| **HapMap replicate** | **Total no. of observed SNPs** | **SNPs correctly observed** | **SNP errors observed** | **Reproducibility Frequency** |
| --- | --- | --- | --- | --- |
| NA18856 | 45081 | 45081 | 0 | 1 |
| NA07000 | 45100 | 45100 | 0 | 1 |
| NA07345 | 45110 | 45110 | 0 | 1 |
| NA11882 | 45127 | 45127 | 0 | 1 |
| NA11993 | 45034 | 45034 | 0 | 1 |
| NA12044 | 45097 | 45097 | 0 | 1 |

**Table S3: Observed replicate consistency using six HapMap individuals.**

Observed IBC version1 array genotyping errors for six replicate HapMap samples, where NA number denotes the official HapMap identifier.
